# Prognostic impacts of left ventricular strain in hemodialytic patients with preserved left ventricular systolic function

**DOI:** 10.1038/s41598-025-97569-0

**Published:** 2025-07-09

**Authors:** Yi-Tsang Fu, Chih-Hsueh Tseng, Wei-Min Huang, Wen-Chung Yu, Hao-Min Cheng, Chern-En Chiang, Chen-Huan Chen, Shih-Hsien Sung, Chih-Ching Lin

**Affiliations:** 1 Department of Medicine, Taipei Veterans General Hospital, Taipei, Taiwan; 2 Department of Medical Education, Taipei Veterans General Hospital, Taipei, Taiwan; 3 General Clinical Research Center, Taipei Veterans General Hospital, Taipei, Taiwan; 4 Cardiovascular Research Center, National Yang Ming Chiao Tung University, Taipei, Taiwan; 5 Department of Internal Medicine, National Yang Ming Chiao Tung University, Taipei, Taiwan; 6 Institute of Emergency and Critical Care Medicine, National Yang Ming Chiao Tung University, Taipei, Taiwan; 7 Department of Medicine, Kinmen Hospital, Ministry of Health and Welfare, Jinhu, Taiwan; 8 Department of Medicine, National Yang Ming Chiao Tung University, No. 155, Sec. 2, Linong Street, Beitou District, 112 Taipei, Taiwan

**Keywords:** Left ventricular global longitudinal strain, Preserved left ventricular ejection fraction, Left ventricular end-systolic volume, Hemodialysis, All-cause mortality, Hospitalization for heart failure, Outcomes research, Cardiology

## Abstract

Left ventricular dysfunction is a known risk factor for morbidity and mortality in hemodialysis patients. The prognostic value of left ventricular global longitudinal strain (LV GLS) among those with preserved left ventricular ejection fraction (LVEF) remains uncertain. Subjects with end-stage renal disease initiated hemodialysis at Taipei Veteran General Hospital between 2015 and 2018 were registered. All participants received annually echocardiographic studies thereafter. Left ventricular end-systolic volume (LVESV), end-diastolic volume (LVEDV) and internal diameter in systole (LVIDs), LVEF, and LV GLS were measured. A LV GLS of > – 15.9% was defined as reduced LV GLS. Clinical outcomes of mortality and hospitalization for heart failure (HHF) were followed. A total of 319 patients with preserved LVEF (66.3 ± 15.1 years, 48.6% men) were recruited in the study. Subjects with reduced LV GLS had more coronary artery disease (CAD), higher LVESV and LVIDs, but were similar in age, gender, co-morbidities, biochemistries and other echocardiographic parameters as the counterpart. Both CAD [(odds ratio (OR) and 95% confidence intervals (CIs): 1.669, 1.023–2.724], and LVESV (OR per-1 mL and 95% CIs: 1.073, 1.004–1.146) were independent determinants of reduced LV GLS. Kaplan-Meier analysis indicated that patients with reduced LV GLS had a significantly lower event-free survival rate compared to those with preserved GLS. The multivariate Cox regression analysis further demonstrated LV GLS as a significant predictor of adverse clinical events (hazard ratio per-1% and 95% CIs: 1.055, 1.002–1.110) after accounting for age, gender, and diabetes. Among the hemodialysis patients with preserved LVEF, LV GLS but not the conventional left ventricular functional indices were associated with long-term mortality and HHF. CAD could be a modifiable risk factor among the subjects with reduced LV GLS.

## Introduction

Patients with end-stage renal disease (ESRD) are associated with increased risks of cardiovascular diseases and heart failure, and their mortality rate is 10 to 20 times higher than that of healthy individuals^1,2^. Numerous data have shown left ventricular systolic or diastolic function is not unusual among subjects with ESRD^2,3^, and there has been an inverse correlation between left ventricular ejection fraction (LVEF) and cardiovascular death or total mortality^4,5^. However, the inverse correlation effect is less pronounced in patients with an ejection fraction greater than 45%^6^. The intrinsic limitations of ejection fraction may restrict its effectiveness in detecting mild left ventricular systolic dysfunction^7^.

Given that LVEF is a volume and load-dependent measure, which varies significantly in subjects with ESRD underwent hemodialysis. In contrast, the two-dimensional speckle tracking echocardiography provides an angle-independent measurement of myocardial deformation, offering a precise evaluation of both systolic and diastolic function across all myocardial segments^8,9^. Left ventricular global longitudinal strain (LV GLS) quantifies the longitudinal shortening of the myocardial fibers^10^, which has outperformed LVEF as a prognostic indicator for adverse clinical events among general population and individuals with heart failure^11,12^.

There is limited data on the utility and prognostic value of strain in the hemodialysis population. Our study aims to explore the determinants and predictors of LV GLS in hemodialysis patients and examine the association between post-hemodialysis LV GLS and clinical outcomes.

## Methods

### Patients

**Fig. 1:**
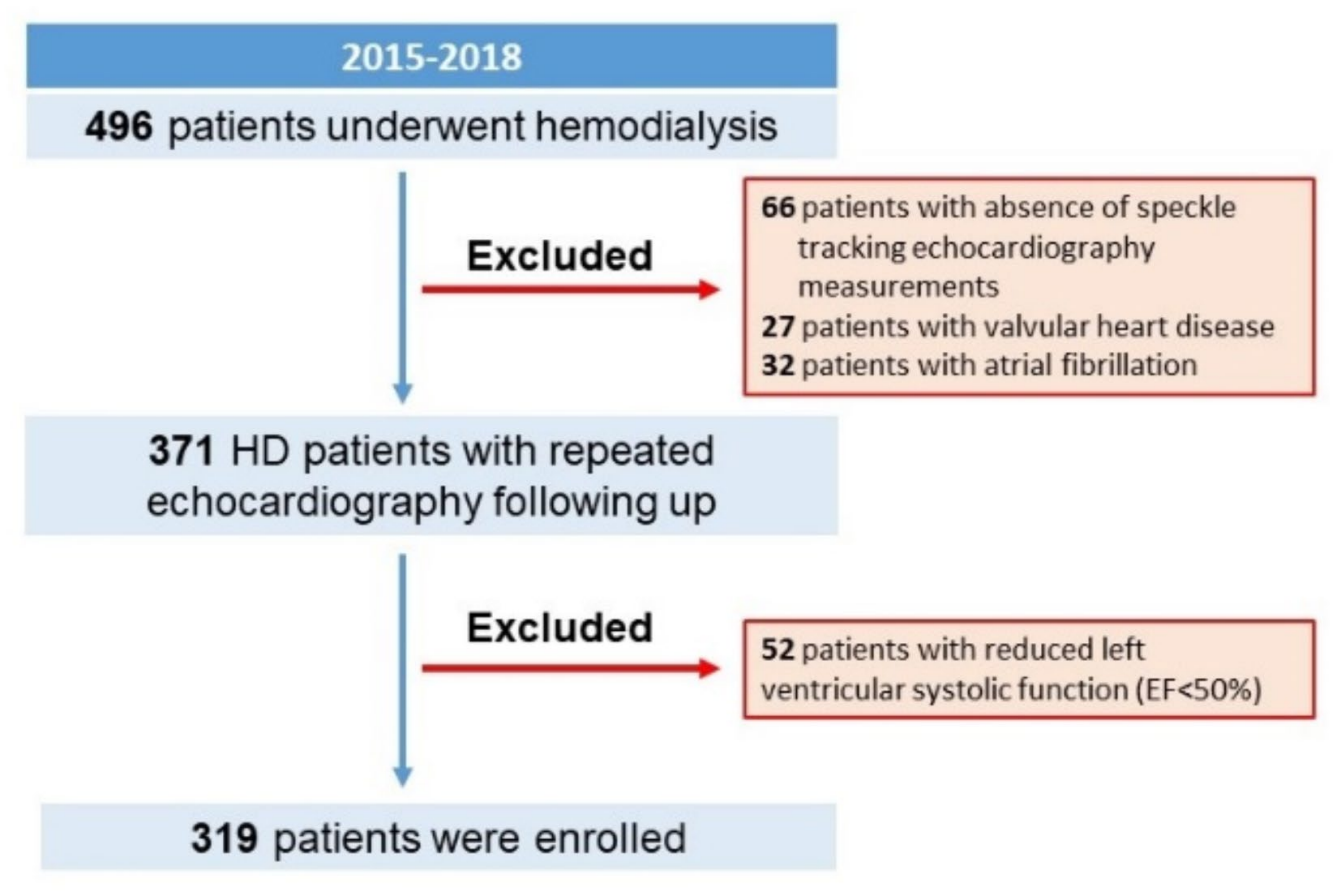
Study subjects enrollment flowchart. From 2015 to 2018, we enrolled 496 patients who began hemodialysis. Several criteria led to the exclusion of some patients. Those who received an echocardiographic evaluation either one month prior or within three months after starting hemodialysis were included. Our study ultimately analyzed a cohort of 319 patients.

From March 2015 to October 2018, patients more than 18 years old receiving chronic hemodialysis for at least 3 months at Taipei Veteran General Hospital were eligible for the present study. The study participants would undergo transthoracic echocardiography annually. Subjects with rhythm other than sinus (*n* = 32), suboptimal image quality for speckle tracking analysis (*n* = 66), significant valvular heart disease (*n* = 27), and LVEF < 50% (*n* = 52) were excluded from this study. The flowchart of the study participants was demonstrated in Fig. 1. The anthropometric and demographic data, and comorbidities were recorded. Hemogram and biochemistries were obtained from the overnight-fasting blood samples, following echocardiographic studies. All subjects provided written informed consent for the use and publication of their clinical data, including anthropometric and demographic details, blood samples, electrocardiograms, and echocardiographic studies. This consent fulfills the study protocol approved by the research ethics committee. The investigation has followed the Declaration of Helsinki with the approval of the Institutional Review Board of Taipei Veteran General Hospital.

### Transthoracic echocardiography

Transthoracic echocardiography was conducted according to the recommendations of the American Society of Echocardiography, utilizing the GE Vivid Untrasound system (Healthcare, Horten, Norway). Left ventricular internal dimensions at end-diastolic (LVIDd) and end-systolic (LVIDs), left ventricular end-diastolic (LVEDV) and end-systolic volume (LVESV), LVEF, early (E) and late (A) ventricular filling flow velocities, the mitral annulus tissue velocity (e’), left atrial volume, and the peak velocity of tricuspid regurgitation (TRV) were measured accordingly^13^. The left ventricular mass index (LVMi), the left atrial volume index (LAVi) and right ventricular systolic pressure (RVSP) were calculated. LVH was defined as LVMi > 95 g/m^2^ for women and > 115 g/m^2^ for men. Left ventricular diastolic dysfunction was defined when ≥ 3 of the following indicators were met: (1) septal e’ velocity < 7 cm/s, (2) septal E/e’ ratio > 14, (3) TRV > 2.8 m/s, and (4) LAVi > 34 ml/m^2^^14^.

Speckle-tracking was conducted across three successive cardiac cycles, capturing two-dimensional left ventricular visuals from the standard triad of apical view. Two-dimensional speckle-tracking assessments were executed by utilizing EchoPAC software (version 10.8, GE Vingmed Ultrasound AS, Horten, Norway). Peak systolic longitudinal strain was evaluated from six segments in apical long-axis, 4-chamber, and 2-chamber perspectives. LV GLS was calculated by averaging regional peak longitudinal strain values captured prior to aortic valve closure, as determined within the apical long-axis field. Longitudinal strain computations were achieved by averaging across eighteen segments: six basal, six mid-ventricular, and six apical segments within the left ventricle^15^. A LV GLS greater than − 15.9% was classified as reduced LV GLS, indicating impaired left ventricular systolic function^16,17^.

### Study endpoints

The adverse clinical events of mortality and hospitalizations for heart failure (HHF) were identified at the outpatient clinics, which was further conformed by reviewing medical recorded and linking the database to the National Death Registry. All the study participants were followed for up to 5 years.

### Statistical analysis

The study population were stratified into preserved and reduced LV GLS. The baseline characteristics were compared by using the chi-square test for categorical variables and the Student’s t-test for continuous variables. The Kaplan-Meier survival curve analysis was conducted to examine the difference in survival probability between groups. The univariate and multivariate logistic regression analyses were utilized to evaluate the determinants of LV GLS. The prognostic values of LV GLS were analyzed by Cox regression analysis. A two-tailed P value of < 0.05 was deemed statistically significant. All statistical analyses were performed using SPSS version 17.0 (IBM, Chicago, IL, USA).

## Results

### Baseline characteristics

A total of 319 patients (age 66.3 ± 15.1 years, 48.6% men) were recruited in this study (Table 1). In general, Age, gender distribution, body mass index, comorbidities, H_2_FPEF score, laboratory tests, and dialytic efficiency were not different between subjects with preserved or reduced LV GLS. But subjects with reduced LV GLS were more likely to have coronary artery disease (CAD). In addition, subjects with reduced LV GLS had greater LVIDs and LVESV, and lower LVEF than those with preserved LV GLS. Otherwise, LVIDd, LVEDV, E and A wave velocity, e’ velocity, E/e’ ratio, LVMi, LAVi, TRV, and RVSP were similar between the two groups.

**Table 1:** Demographic, clinical, and echocardiographic variables in Hemodialysis patients with preserved LV GLS or reduced LV GLS.

	Hemodialysis population, *n* = 319			
	Total population	Preserved LV GLS, *n* = 197	Reduced LV GLS, *n* = 122	p-value
Demographics				
Age, years	66.33 ± 15.13	66.44 ± 14.57	66.16 ± 16.03	0.876
Male Gender, n (%)	155 (48.6%)	90 (45.7%)	65 (53.3%)	0.187
BMI, Kg/m^2^	24.19 ± 3.72	24.10 ± 3.94	24.33 ± 3.36	0.565
Comorbidities				
Hypertension, n (%)	230 (72.1%)	141 (71.6%)	89 (73%)	0.790
Diabetes mellitus, n (%)	163 (51.1%)	95 (48.2%)	68 (55.7%)	0.192
Coronary artery disease, n (%)	109 (34.2%)	58 (29.4%)	51 (41.8%)	**0.024**
H_2_FPEF score	2.58 ± 1.40	2.55 ± 1.42	2.64 ± 1.38	0.600
Laboratory tests				
Hb, g/dL	10.18 ± 1.18	10.13 ± 1.08	10.25 ± 1.32	0.385
eGFR, mL/min/1.73 m2	7.27 ± 5.26	6.82 ± 4.12	8.12 ± 6.73	0.072
Albumin	3.71 ± 0.38	3.74 ± 0.35	3.67 ± 0.43	0.106
Ca, mg/dL	8.72 ± 0.97	8.98 ± 0.89	8.92 ± 0.82	0.488
P, mg/dL	5.11 ± 1.48	5.02 ± 1.44	5.25 ± 1.55	0.192
Dialysis efficiency				
Dialyzer flow, ml/min	252.08 ± 32.24	253.27 ± 33.54	250.16 ± 30.07	0.403
Dialysis duration, hr	3.91 ± 0.22	3.92 ± 0.21	3.91 ± 0.23	0.755
Ultrafiltration, kD	2.34 ± 1.09	2.40 ± 1.17	2.26 ± 0.98	0.259
Echocardiographic parameters				
LVIDd, cm	4.86 ± 0.69	4.84 ± 0.67	4.89 ± 0.76	0.486
LVIDs, cm	3.03 ± 0.56	2.97 ± 0.52	3.14 ± 0.61	**0.017**
LVEDV, mL	80.16 ± 28.06	78.00 ± 25.16	83.65 ± 32.02	0.099
LVESV, mL	33.09 ± 13.21	31.65 ± 11.63	35.43 ± 15.21	**0.020**
LVEF, %	59.04 ± 4.77	59.67 ± 4.86	58.03 ± 4.48	0.134
E, cm/s	89.90 ± 29.54	88.42 ± 27.45	92.37 ± 32.70	0.258
A, cm/s	104.84 ± 25.39	105.56 ± 24.21	103.60 ± 27.41	0.525
E/A ratio	0.88 ± 0.38	0.86 ± 0.35	0.93 ± 0.44	0.192
Septal e’, cm/s	6.50 ± 1.73	5.39 ± 1.68	5.44 ± 1.50	0.801
Septal E/e’ ratio	17.56 ± 6.66	17.28 ± 5.95	18.04 ± 7.73	0.348
LAV index, mL/m^2^	56.13 ± 9.32	55.97 ± 8.37	56.39 ± 10.72	0.694
TR velocity, cm/s	268.1 ± 51.25	265.91 ± 51.22	272.54 ± 51.26	0.273
RVSP, mmHg	35.66 ± 12.73	34.91 ± 12.34	36.90 ± 13.32	0.188
LVM index, g/m^2^	151.23 ± 45.61	149.93 ± 46.24	153.32 ± 44.66	0.521
LVH	280 (87.7%)	173 (87.8%)	107 (87.7%)	0.953
Diastolic dysfunction, n (%)	107 (37.9%)	62 (34.4%)	45 (44.1%)	0.108
Global longitudinal strain parameters				
AP4L strain, %	− 18.72 ± 4.47	− 19.44 ± 3.93	− 17.57 ± 5.04	**0.001**
AP2L strain, %	− 19.13 ± 5.31	− 19.63 ± 5.84	− 18.34 ± 4.22	**0.035**
AP3L strain, %	− 18.24 ± 5.62	− 18.86 ± 5.44	− 17.26 ± 5.79	**0.013**
LV GLS, %	− 18.35 ± 4.98	− 18.84 ± 5.34	− 17.56 ± 4.26	**0.001**

Significant values are in [bold].

AP4L: Apical four-chamber longitudinal strain; AP2L: Apical two-chamber longitudinal strain; AP4L: Apical three-chamber longitudinal strain; A = Mitral late-diastolic inflow peak velocity; BMI = Body mass index; Ca = Calcium; E = Mitral early-diastolic inflow peak velocity; E/A ratio = Mitral Ratio of Peak Early to Late Diastolic Filling Velocity; eGFR = estimated Glomerular filtration rate; Hb = Hemoglobin; LV LV GLS = Left ventricular global longitudinal strain; LVH = Left ventricular hypertrophy; LVM = Left ventricular mass; LVIDd = End diastole left ventricular internal diameter; LVIDs = End systole left ventricular internal diameter; LVEDV = Left ventricular end-diastolic volume; LVESV = Left ventricular end-systolic volume; LVEF = Left ventricular ejection fraction; LAV = Left atrial volume; P = Phosphorus; RVSP = Right ventricular systolic pressure; Septal E/e’= Septal early trans-mitral velocity to tissue Doppler mitral annular early diastolic velocity ratio; TR velocity = Peak velocity of tricuspid regurgitation.

In contrast, subjects with preserved LV GLS truly had greater apical longitudinal strains, including AP4L strain, AP2L strain, and AP3L strain, as well as LV GLS than their counterpart.

### Predictors of reduced LV GLS

CAD, LVIDs, and LVESV were crudely associated with the presence of reduced LV GLS. In the multivariate logistic regression analysis, CAD [(odds ratio (OR) and 95% confidence intervals (CIs): 1.669, 1.023–2.724], and LVESV (OR per-1 mL and 95% CIs: 1.073, 1.004–1.146) remained related to reduced LV GLS. (Table 2)

**Table 2:** Predictors of reduced left ventricular global longitudinal strain.

Predictive variables	Univariate		Multivariate	
	OR (95% CI)	p-value	OR (95% CI)	p-value
Age	0.999 (0.984–1.014)	0.875	0.997 (0.981–1.013)	0.684
Male Gender	0.738 (0.469–1.160)	0.188	0.870 (0.521–1.453)	0.627
DM	1.352 (0.859–2.128)	0.192	1.239 (0.771–1.989)	0.376
CAD	1.721 (1.073–2.761)	**0.024**	1.669 (1.023–2.724)	**0.040**
LVIDs, cm	1.676 (1.111–2.529)	**0.014**	1.296 (0.766–2.190)	0.334
LVEDV, mL	1.007 (0.999–1.015)	0.083	0.972 (0.943–1.001)	0.061
LVESV, mL	1.022 (1.004–1.040)	**0.015**	1.073 (1.004–1.146)	**0.039**

Significant values are in [bold].

CAD = Coronary artery disease; DM = Diabetes mellitus; LVIDs = End systole left ventricular internal diameter; LVEDV = Left ventricular end-diastolic volume; LVESV = Left ventricular end systolic volume.

### Prognostic values of LV GLS

**Fig. 2:**
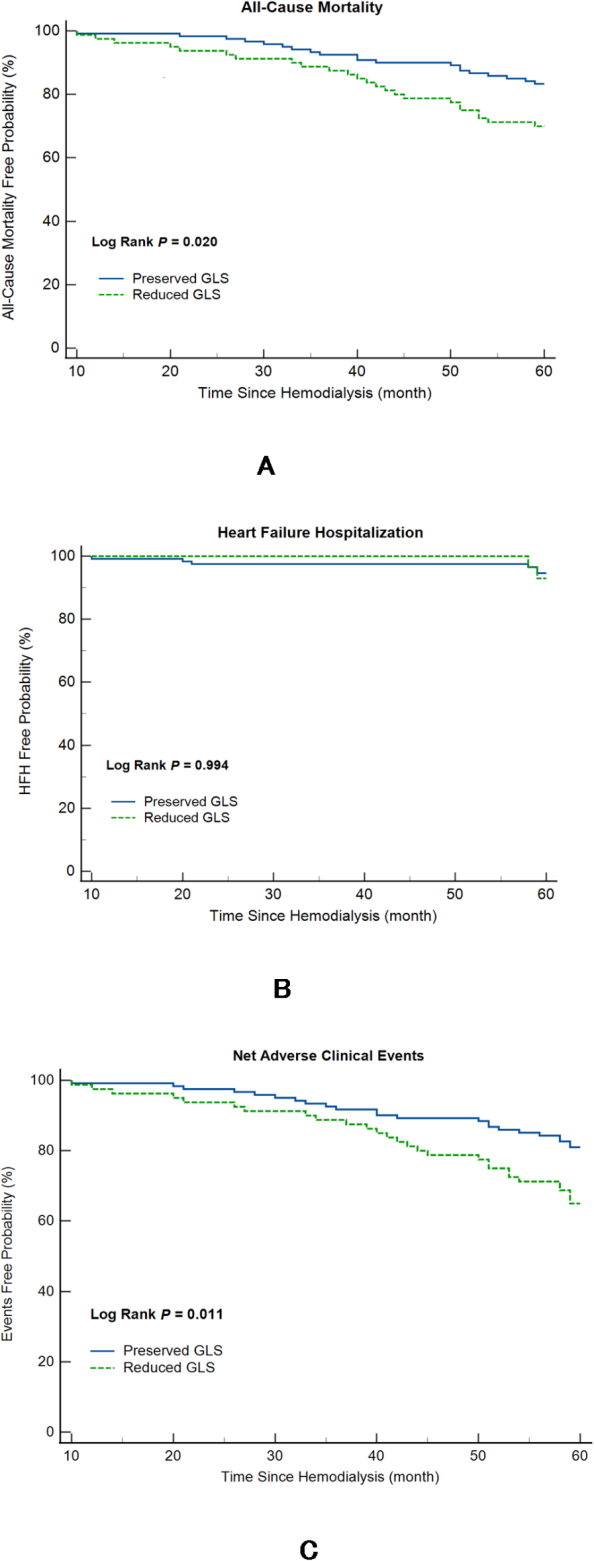
Difference of outcomes in improved GLS and reduced GLS hemodialysis patients. Through Kaplan-Meier analysis, a deterioration in left ventricular (LV) global longitudinal strain (GLS) was substantially correlated with an increase in net adverse clinical events and all-cause mortality rates.

During a mean follow-up period of 4.7 years, there were 44 (13.7%) deaths and 10 (3.1%) HHF across the entire cohort. Among patients with preserved LV GLS, 23 (11.6%) of them encountered HHF or mortality, while 28 (22.9%) of those with reduced LV GLS had adverse clinical outcomes. The Kaplan-Meier survival curve analysis showed better event-free survival among subjects with preserved LV GLS than the others (Fig. 2A), which was mainly driven by the lower risk related to preserved LV GLS (Fig. 2B). However, the rates of HHF were not different between patients with reduced and preserved LV GLS (Fig. 2C).

**Fig. 3:**
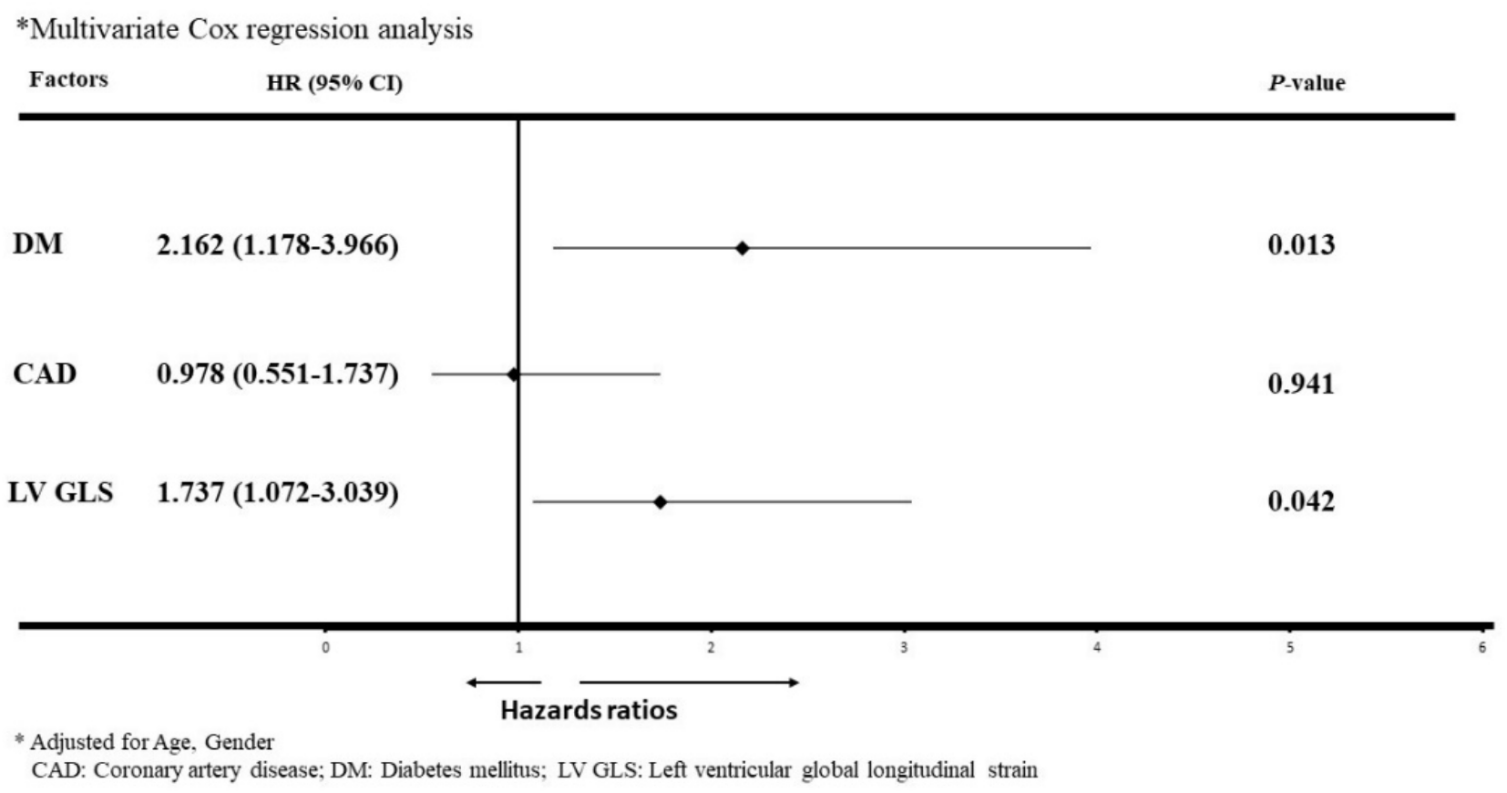
COX- regression analysis of predictors of Overall Events in hemodialysis patients. After performing a multivariate Cox regression analysis, the left ventricular global longitudinal strain (LV GLS) remained a significant predictor of Net Adverse Clinical Events rates in hemodialysis patients.

In a multivariate Cox regression model, presence of diabetes and reduced LV GLS were all independently predictive of worse outcomes in subjects with ESRD and preserved LVEF (Fig. 3). None of the other measures of LV structure or function, including LVIDs, LVEDV, LVESV, LVEF, and diastolic dysfunction correlated with the clinical outcomes. With adjustments for age, gender, and diabetes, LV GLS (HR per-1% and 95% CIs: 1.055, 1.002–1.110) remained significant predictors of adverse clinical events (Table 3).

**Table 3:** COX-regression analysis of predictors of net adverse clinical events in Hemodialysis patients.

Predictive variables	Univariate		*Multivariate	
	HR (95% CI)	p-value	HR (95% CI)	p-value
Septal E/e’ ratio	1.029 (0.991–1.069)	0.136	1.012 (0.966–1.061)	0.610
LVIDs, cm	1.071 (0.661–1.735)	0.780	1.308 (0.776–2.205)	0.314
LVEDV, ml	0.998 (0.988–1.008)	0.642	1.001 (0.991–1.012)	0.805
LVESV, ml	1.001 (0.978–1.023)	0.991	1.007 (0.985–1.031)	0.529
LVEF, %	0.956 (0.889–1.016)	0.147	0.963 (0.904–1.027)	0.252
Diastolic dysfunction	1.376 (0.751–2.522)	0.302	1.042 (0.565–1.921)	0.896
LV GLS, %	1.751 (1.572–1.930)	**0.037**	1.055 (1.002–1.110)	**0.043**

Significant values are in [bold].

*Adjusted for age, gender, DM.

DM = Diabetes mellitus; LVIDs = End systole left ventricular internal diameter; LVEDV = Left ventricular end-diastolic volume; LVESV = Left ventricular end systolic volume; LVEF = Left ventricular ejection fraction; LV GLS: Left ventricular global Longitudinal Strain; Septal E/e’= Septal early trans-mitral velocity to tissue Doppler mitral annular early diastolic velocity ratio.

## Discussion

The study investigated the prognostic roles of left ventricular function in subjects with ESRD receiving chronic hemodialysis. The results demonstrated LV GLS but not LVEF or diastolic dysfunction was associated with long-term risks of mortality and HHF. In addition, CAD and LVESV were independent determinants of reduced LV GLS. Consequently, the results may underscore the importance of using LV GLS for clinical risk evaluation among hemodialytic patients who deem had preserved LVEF.

LV GLS has been a sensitive discriminator over clinical risk factors in predicting all-cause mortality in patients with renal impairment and preserved LVEF^18^. In a prospective cohort study of 88 stable hemodialysis patients, reduced LV GLS was independently associated with mortality during a mean follow-up duration of 26 months^19^. The present study further extended the long-term prognostic impact of LV GLS on mortality and HHF. Therefore, abnormal LV GLS could serve as an early indicator of uremic cardiomyopathy in dialysis patients when they have normal LVEF. The underlying causes of abnormal LV GLS in ESRD may involve the interstitial fibrosis with myocyte hypertrophy^20^. In animal models of uremic cardiomyopathy, Rafael et al. illustrated that strain parameters can detect uremic cardiomyopathy and predict cardiovascular mortality among dialysis patients^21^. Although there were only minor differences in left ventricular structures and functions in this study, LV GLS outperformed other cardiac parameters in the prediction of adverse clinical events. The study results may support LV GLS a favorable index of uremic cardiomyopathy.

LVH is commonly observed among subjects with ESRD, and left ventricular chamber size usually would be small, especially in patients with normal LVEF. Even though, Wu et al. have highlighted LVIDs, but not LVEF an independent risk factor of major adverse cardiovascular events and mortality in chronic hemodialysis patients^22^. The dependability of LVEF as a functional index is affected by its sensitivity to external elements like preload, afterload, and heart rate^23^. Based on current understandings, LVEF may be more reflective of LV dilation than myocardial contractility^24,25^. In contrast, LVESV is less susceptible to cardiac loading conditions and more responsive to variations in contractility, indicating LVESV a better index of left ventricular systolic function^26,27^. In this study, 87.7% of the population had LVH and left ventricular chamber size were all within normal values. LVESV was a sensitive index related to LV GLS and LVEF, underscoring left ventricular chamber size for risk stratifications^28–30^. However, none of LVIDs, LVESV or LVEF was associated with the outcomes.

Increased pressure, volume overload, anemia, hypocalcemia, and hyperphosphatemia may have explain diastolic dysfunction but not systolic dysfunction was more prevalent in patients with ESRD^31–33^. Among patients with HFpEF, LV GLS has been associated with left ventricular end-diastolic pressure, indicating that a decrease in LV GLS may precede early-stage LV diastolic dysfunction^14^. In this study, we did not demonstrated significant association between diastolic dysfunction and LV GLS, neither the diastolic dysfunction correlated with the outcomes^34^. The results may suggest LV GLS a better indicator of left ventricular abnormalities beyond systolic and diastolic dysfunction.

The study also demonstrated CAD as a key determinant of LV GLS in hemodialysis patients. This could be attributed to the susceptibility of the endocardium to the adverse effects of reduced blood flow or ischemia. Given that the endocardial myofibers predominantly have a longitudinal orientation, LV GLS tends to deteriorate quickly under ischemic conditions^35^. The results may alert the clinical physicians to survey for CAD among subjects with reduced LV GLS but normal LVEF. However, the clinical benefits of subsequent coronary interventions needs further investigations.

Since conventional echocardiographic parameters are related to volume fluctuations, there’s a concern about the impact of fluid load on LV GLS in patients undergoing hemodialysis. Liu et al. have shown the measures of LV GLS during hemodialysis or on an interdialytic day were consistent^9^. Moreover, recent researches have indicated that LV GLS is considered to be less influenced by both pre-load and after-load^36,37^.

Given the nature of an observational study, there were inherent limitations. Firstly, the study demonstrated LV GLS did not correlate with HHF because of the relatively small size of the study cohort and limited events. Therefore, we may not have sufficient statistical power to exclude the prognostic association of traditional left ventricular parameters. Secondly, we did not assess the levels of brain natriuretic peptide (BNP), while BNP has its clinical utility in ESRD, it doesn’t offer the comprehensive cardiac insights provided by echocardiography for these patients.

## Conclusion

Among asymptomatic hemodialysis patients with preserved LVEF, we demonstrated LV GLS was independently associated with 5-year mortality and HHF. None of the other indices, including left ventricular chamber sizes, LVEF and diastolic function correlated with the clinical outcomes. In addition, CAD and LVESV were major determinants of LV GLS. The study results may encourage the implement of LV GLS as a routine measure in hemodialysis patients to early detect high-risk subjects even with normal LVEF. And the further survey for CAD among those with reduced LV GLS may offer opportunities to improve prognosis when coronary intervention is indicated.

## Data Availability

The datasets generated and analysed during the current study are not publicly available due to patient privacy concerns and legal restrictions associated with the confidentiality of medical records. However, they are available from the corresponding author on reasonable request.

## Abbreviations

LV GLS: Left ventricular global longitudinal strain
LVESV: Left ventricular end-systolic volume
